# A Clinical Lecture on Rheumatic Pericarditis

**Published:** 1906-07-14

**Authors:** J. A. Nixon

**Affiliations:** Assistant Physician to the Bristol Royal Infirmary.


### Hospital Clinics.

### A CLINICAL LECTURE ON RHEUMATIC PERICARDITIS.

By J. A. Nixon, M.B. (Cantab), M.R.C.P., Assistant Physician to the Bristol Royal Infirmary.

There are few diseases which can be so easily recognised by even the incompetent observer from the classical description as given in any text-book than pericarditis, provided the conditions present conform to the type described, but it is also true that these conditions are so commonly atypical that with increased clinical experience and the acquisition of greater skill in observation there comes a growing conviction of the likelihood of the complication when it occurs in acute rheumatism going undetected. The most unequivocal physical signs and symptoms are precisely those most frequently absent in a particular case, and the less definite indications may prove on closer study to be of commoner occurrence.

Pericarditis, as a complication of acute rheumatism, shows a remarkable tendency to occur in "epidemics"; in an extended series of cases of rheumatism the complications are apt to appear in groups; one set of cases is characterised by the acuteness of the fever and severe inflammation of the joints, yet the patients escape without any affection of the heart; while on another occasion all the cases appear to develop valvular lesions, either with or without the fever and joint manifestations being very pronounced; while in yet other groups pericarditis seems to be the rule. What governs these variations in the behaviour of the disease remains for the present an unsolved mystery, and the phrase of Sydenham's, "The epidemic constitution of disease," is one which we are no more able to explain than entitled to reject. Some help in diagnosing the condition may be gained from recognising its liability to occur in periodic groups.

It becomes usual from time to time to state that pericarditis and pericardial effusion are less commonly met with than formerly, but the observation is difficult of proof: it is true that fewer cases of rheumatic fever end fatally now than in the days when blood-letting was the universal treatment and before salicylates had been introduced, so that the discovery of pericarditis is less often made in the post-mortem room, and Sir Thomas Watson's dictum remains full of force that "the diagnosis of pericarditis has been confessedly uncertain and obscure." Yet on the whole, though the incidence of pericarditis is probably not less frequent, its recognition *may* be, and its verification by autopsy undoubtedly *is* rarer.

### Diagnosis of Pericarditis.

The differentiation of cardiac inflammations into endo-, myo-, and peri-carditis, if it can be admitted at all, can only extend to the severer cases, and that only so far as the physical signs, and not the pathological processes, are considered; it must be allowed at once that no inflammation of the pericardium which gives rise to physical signs can be conceived in which the myocardium is not involved, and in which, it may be, the endocardium also suffers; the converse may also be considered as at least likely that a severe endocarditis cannot with certainty be dissociated from inflammatory changes in the other integral parts of the cardiac system. So that while agreeing to consider the complication of pericarditis as a thing apart, we shall at least be in good company if we admit the more exact term for all these inflammations to be rheumatic carditis, for so Sir Thomas Watson insisted they should be set down.

There is a group of cases, however, in which the pericardium is, at least apparently, mainly involved, and it is this class with which we are at present concerned. The primary attack is usually an event of childhood; it is to children in their first attack of rheumatic fever that the first attack of pericarditis comes: adults are less commonly the subjects of a first attack of pericarditis in the same degree as they are less often seized with acute rheumatism when childhood is past.

The symptoms of pericarditis are always in advance of the physical signs, but, as Latham pointed out, we may overlook pericarditis either because the signs or the symptoms are absent; if we are on our guard and realise this the diagnosis will probably more often be made correctly; it is in very few cases "that pericarditis coming on just at the suspected time and just under the suspected circumstances could *now* be overlooked."

What, then, is to be looked for so anxiously? What is it that "every prudent physician searches after day by day in all cases of acute rheumatism"?

The general symptoms have been inimitably described: "There is often a singularity of manner and peculiar expression of countenance, difficult to describe, yet strikingly manifest to the observer, a strangeness of deportment mixed somehow with an aspect of distress. To this are frequently added pulsation, a sense of fullness in the epigastrium a catch in the breathing, a dry cough, inability or unwillingness on the part of the patient to lie on his left side; pain in the situation of the heart, increased by a full inspiration, by pressure upon or beneath the corresponding ribs, and more particularly increased by pressure upwards against the diaphragm by means of the fingers thrust beneath the cartilages of the false ribs; stiffness and pain in and about the left shoulder, and extending thence down the left arm, and stopping short, perhaps, at the elbow or wrist. This last circumstance, however, the pain shooting down the arm is more common in *chronic* affections of the heart. And I have yet another symptom to mention, and a very important one; and that is, delirium, sometimes quiet, but of the wild and furious delirium, not dependent upon any disease of the encephalon."

The description is from a book that is now old and perhaps too little read, but it conjures up pictures of real patients one has seen, and requires no elaboration to obscure its precision.

### The Signs of Pericarditis.

It is convenient in text-book description to allot certain physical signs to certain stages in the disease, at the same time it is of small value in the observation of any particular case to pin too much faith upon any such arbitrary divisions; what concerns the patient most intimately is the manner in which his heart discharges its functions, not the imaginary pictures of the external coverings of that organ. To fail to diagnose with absolute certainty the existence of pericardial inflammation is of less importance to the individual patient than to disregard the slenderer clues of heart failure from whatever cause: the safe physician is not anxiously noting the fleeting signs and symptoms in order that his opinion may be justified at the autopsy, but rather that the autopsy may be avoided. But to be safe, the complication must be suspected before and recognised at its outset.

The signs of pericarditis divide themselves into two classes, those which are fairly constant and those which are definitely inconstant; and one of our difficulties is that the more constant signs are not peculiar to the condition, but are shared by many of the cardiac complications of rheumatic fever.

There are three signs, then, which may be confidently expected in the earliest stages of pericarditis:—Pyrexia, Increased frequency and irregularity of the heart-beat, Failure of the first sound of the heart.

*Pyrexia.*—This sometimes takes the form of a sudden rise in the temperature, but is usually only observed to vary with great suddenness or through two or three degrees if the temperature of the original fever had returned to nearly normal; the more common way in which the temperature attracts attention is that, as the joint lesions subside and we look for a fall in the temperature to correspond, there is a persistence in, or even increase of, the fever.

*Pulse.*—At first a trifling irregularity is detected at the wrist, a beat dropped, an undue pause between the beats, or a short series of two or three running beats; later there is a definite increase in the pulse rate, or—and it is by no means the same thing in pericarditis—in the frequency of the heart's action, for the impulse may very often fail to reach the wrist.

*Heart Sounds.*—Although the changes which may take place in the quality of the heart sounds are innumerable, there is one which will be found on practically every occasion; that is, the failure of the first sound. This sound is very early altered by any active disease of the heart, and it is always so affected that it loses its definition; it ceases to have the clear, vigorous "thump" of health; it becomes muffled, longer drawn out, or ceases to be heard at all.

When this is observed it is all-sufficient for the patient, his heart is failing to contract in its usual way; for one reason or another it is "not up to form." If we realise that the patient has rheumatic carditis there is no fear of making a serious mistake in waiting for the appearance of one of the many inconstant signs to give information as to the particular variety of the carditis.

Of the inconstant signs those more frequently met with are:—The cantering rhythm of the heart; friction—at first systolic in time, later "to and fro"; Displacement of the apex beat; Increase of the area of cardiac dullness.

*Cantering or triple rhythm.*—This phenomenon is sufficiently characteristic to be easily recognisable when once heard; it is probably better described as a re-duplication in the first heart sound; it has been phonetically rendered as "ti-ti-tup," and perhaps represents a halting or hesitating contraction of the ventricles. When met with in the course of acute rheumatism, watch for other signs of pericarditis.

*Friction.*—A pericardial rub is far from uncommon at some stage of pericarditis, whether it is easy to make sure of its real nature is quite another matter. In most cases it is at first systolic in time—very like an endocardial murmur, but scarcely so constant in its relation to the first sound, and it is by this variability in its "time relations" that it may be recognised. Of course, when the friction amounts to a "rub" like sand-paper, or becomes "to and fro" in rhythm it is easily known; the only precaution necessary being to exclude a pleural or pulmonary origin for the sound, which may in most cases be done by making the patient hold his breath during auscultation. When a wooden stethoscope is used (and none other is reliable in dealing with the heart) a pericardial sound may be distinguished from an endocardial murmur by the fact that on relaxing the pressure of the ear upon the stethoscope the heart sounds can still be heard without the pericardial, but in the case of an endocardial murmur it can be heard as far as the heart sounds are distinguishable. Early friction is oftenest heard near the base of the heart. Remember, pericardial friction does not disappear when an effusion develops, like pleural friction.

*Apex Beat.*—The apex-beat is almost always displaced at some time, but it is impossible to be dogmatic as to the direction of this displacement. When the first signs of heart failure occur the apex-beat will be displaced outwards, and as the left side of the heart dilates it will move further outwards and downwards.

If there is effusion, and that effusion happens to encroach upon the anterior aspect of the heart, the visible or palpable pulsation may be displaced inwards, being no longer due to the impulse of the true apex, but rather of some other portion of the heart nearer to its widest circumference. And beyond this point the area of cardiac dullness will frequently extend to the left.

*Area of Cardiac Dullness.*—An increase in this area may take place from two causes in pericarditis, either from the dilatation of the heart or from a distension of the anterior aspect of the pericardium with fluid. In the former case the increase will be chiefly outwards, partly to the left, and as the right heart suffers, to the right, but practically never upwards. This is the direction which is almost the peculiar province of a pericardial effusion, and if an increase of this nature is found it is of the utmost importance, but many large effusions never collect in this part of the pericardial sac, and the absence of such an increase in the dullness is no proof that a pericardial effusion does not exist.

Finally, there is a group of occasional signs, such as may be present and give great assistance in the diagnosis, but whose absence is of no importance:—Pulmonary and pleural signs; some special decubitus; Excessive sweating; Friction-fremitus or thrill; Effects of posture on the cardiac impulse.

*Decubitus.*—Almost every variety of posture may be met with in pericarditis; the majority of patients certainly prefer the semi-recumbent position; few, if any, can lie comfortably on either side. Beyond this one meets with an occasional case where the patient lies flat on the back, refusing even a pillow for the head; and others, again, are only at ease when leaning forward slightly, and like to rest their arms on a bed-table. There are no general conclusions to be drawn from a definite posture.

*Effects of Change of Posture.*—These may be important and helpful in a doubtful case of pericardial effusion.

The cardiac impulse may be imperceptible in the recumbent position, but becomes evident when the patient sits up or bends forward. The area of cardiac dullness may be increased by sitting up or bending forwards.

The relative intensity of the cardiac sounds or murmur may be influenced by change of position.

*Friction-Fremitus.*—In a small proportion of cases with extensive friction a distinctive thrill or rub may be palpable to the hand over the precordium.

*Excessive Sweating*, especially about the face, is often noted in patients with pericarditis, after the general and characteristic perspiration associated with rheumatic fever has subsided.

*Pulmonary and Pleural Signs.*—The effects of a large pericardial effusion upon the neighbouring structures, and especially the lungs, are often marked, but vary greatly in different cases. Of the more constant may be mentioned dullness in the left axillary base, sometimes extending round to the back, and the signs of compression of the lung close to the angle of the scapula, shown by a small area of dullness, increased vocal fremitus, bronchial breathing and bronchophony, but without crepitations.

But that these signs do not always depend upon the merely mechanical effects of a distended pericardium is evidenced by the frequency with which effusion is met with in one or both pleuræ.

### Treatment.

The main indications in treating pericarditis are to ensure rest to the patient and rest to the heart.

Naturally then the patient is kept in bed, and in the most comfortable position; movement is avoided as far as possible, and this should be kept in mind in ordering aperients; they should be calculated to disturb the patient as little as possible, enemata are best, provided they can be administered with skill. Local applications, as Leeches, Blistering, application of the ice-bag, Mustard-leaves, or Linseed meal and bran poultices will all in their turn prove useful. Whether they have any active power of arresting the inflammation is a point vigorously debated by the advocates of any individual one of these remedies. Certainly in different cases they have each or all at some time a beneficial and soothing effect upon particular patients.

Drugs.—Salicylates may, as a rule, be continued with apparent advantage, and if they fall short of our expectations the substitutes, such as *Aspirin*, Salicin, Salophen, etc., may be given a trial; more important, however, than their use is their intelligent discontinuance if they appear, as they often do, to have a depressant effect on the patient.

Delirium and pain are best treated with opium; and for some reason Dover's powder appears to be the most satisfactory form in which to give it. The after-effects of the opium are less unpleasant, and the drug can be tolerated in repeated doses over a considerable length of time. For adults grains x. and for children grains ij. to v. may be ordered three times a day, though it is only the severest cases that will require it more than once a day, towards evening. The tincture of opium is the next best form to administer. Morphia does not seem quite as reliable, but a hypodermic injection of $\frac{1}{6}$ to $\frac{1}{4}$ grain may act rapidly if the pain and distress become suddenly acute in adults; for children hypodermic injections are totally unsuitable.

It is worth noticing that the combination of strychnine with the morphia often produces a greater sedative effect in cardiac restlessness than the morphia alone, and that in many cases where the symptom is general distress rather than pain, the strychnine by itself acts more quickly and certainly.

Inhalations of oxygen (for five minutes every hour) sometimes have a most welcome hypnotic effect in children when there are signs of cardiac failure.

Chloral, chloralamide, and paraldehyde and similar hypnotic drugs can at best be said only to be occasionally efficacious; they are always worth a trial, however, for patients show remarkable idiosyncrasies in their appreciation for and reaction to sedatives.

Always watch for respiratory embarrassment, and treat accordingly. Ammonium carbonate (gr. iij. v. in milk) is the best remedy.

Stimulants.—It is a mistake to regard some kind of stimulant as necessary from the first; it may be more urgently required later.

Restlessness, cyanosis, irregularity of the heart's action, and failure of the pulse to reach the wrist are the leading indications for stimulants, and a slow pulse may occasionally be of more serious import than a rapid. Strychnine and alcohol are the only stimulants worth giving unless the old-fashioned musk is resorted to, when a drachm of the tincture is better administered by mouth than many of the more newly invented hypodermic remedies.

*Paracentesis pericardii.*—This operation is now but rarely resorted to; its value is very doubtful: possibly an inflamed heart is less impeded in its action when beating in a sac full of fluid than when rubbing against an inflamed pericardium; at least after the pericardium has been tapped the patient is occasionally very uncomfortable until the fluid has reaccumulated. Anterior puncture in the various interspaces that have been recommended usually leads to the needle finding the heart, not the fluid.

Of the prognosis it may be said that, excepting young children, few patients die of acute rheumatic pericarditis, and few fail to die of its after-effects.

A patient may be expected to recover from the immediate attack with a heart so damaged that it will inevitably cause death at some subsequent time.

